# Low level of Cyclin‐D1 correlates with worse prognosis of clear cell renal cell carcinoma patients

**DOI:** 10.1002/cam4.2313

**Published:** 2019-06-10

**Authors:** Qing‐shui Wang, Feng Li, Zi‐qiang Liao, Ke Li, Xin‐liu Yang, You‐yu Lin, Yi‐lin Zhao, Shu‐yun Weng, Yun Xia, Yan Ye, Su‐huan Li, Chen‐yi Wang, Yao Lin

**Affiliations:** ^1^ Provincial University Key Laboratory of Cellular Stress Response and Metabolic Regulation, Key Laboratory of Opto Electronic Science and Technology for Medicine of Ministry of Education, College of Life Sciences Fujian Normal University Fuzhou P.R. China; ^2^ Department of Pathology Provincial Clinical Medical College of Fujian Medical University Fuzhou P.R. China

**Keywords:** clear cell renal cell carcinoma, Cyclin‐D1, grade, prognosis, recurrence

## Abstract

Cyclin‐D1 (CCND1) belongs to the highly conserved cyclin family whose members are characterized by abundant expression during the cell cycle. As an oncogene, high level of CCND1 was observed and related to poor prognosis and tumor recurrence in many cancers. In this study, we focused on the role of CCND1 in the clinical outcome of clear cell renal cell carcinoma (ccRCC). Gene Expression Omnibus database, The Cancer Genome Atlas database, and immunohistochemical staining were used. The mRNA and protein levels of CCND1 were significantly enhanced in ccRCC tumor tissues. However, the low level of CCND1, but not high level of CCND1, was related to poor prognosis and tumor recurrence in ccRCC. Further analysis showed that CCND1 mRNA level decreased with increasing ccRCC tumor grades and the rate of recurrence in ccRCC patients. In a nomogram model, the CCND1 mRNA level was shown to help predict ccRCC patient recurrence. CCND1 is a strong determinant for prediction of recurrence. The patients with high CCND1 level appear to have a more favorable prognosis together with more frequent low‐grade tumors and low rate of recurrence. This is the first study to investigate the prognostic roles of CCND1 in ccRCC and discovered that CCND1 had an unconventional positive impact on the clinical outcome of ccRCC patients.

## 1 INTRODUCTION

Renal cell carcinoma (RCC) accounts for more than 80% of kidney cancer and represents about 3% of adult malignant tumors.^1^ RCC contains multiple pathological categories including chromophobe RCC (cRCC), clear cell RCC (ccRCC), and papillary RCC (pRCC). Hypertension, history of smoking, and obesity are the three known risk factors for RCC.^2^ Previous studies have estimated that about 20%‐40% of RCC patients may experience recurrence after therapy.^3^ It has been reported that tumor size, tumor grade, stage, and microvascular invasion are related to the disease progression or recurrence of RCC.^4^, ^5^, ^6^, ^7^ ccRCC, the most common form of renal cancer, accounts for 80%‐90% of all diagnoses.^8^ However, the molecular biomarkers associated with recurrence of ccRCC have not been well established. There is an urgent need for new biomarkers to predict the risk of recurrence for ccRCC patients.

Cyclin‐D1 (CCND1) is a protein required for progression through the G1 phase of cell cycle.^9^ During the G1 phase, CCND1 is synthesized quickly and accumulates in the nucleus and is degraded when the cell enters the S phase.^9^ CCND1 regulates the G1/S phase transition by dimerizing with cyclin‐dependent kinase 4 (CDK4)/CDK6. The CCND1‐CDK4 complex promotes the G1/S phase transition by inhibiting retinoblastoma protein (Rb) through phosphorylation and allows E2F transcription factors to transcribe genes required for entry into the S phase.^10^ CCND1 is a well‐recognized oncogene and overexpressed in a considerable proportion of human cancers including breast carcinoma, bladder cancer, pituitary adenomas, head and neck squamous cell carcinomas, pancreatic carcinomas, and non‐small‐cell lung cancers.^11^, ^12^, ^13^, ^14^, ^15^, ^16^ Previous studies have shown that higher CCND1 level is associated with increased recurrence of multiple cancers including giant cell tumor of bone, squamous cell carcinoma of the tongue, and supratentorial ependymomas.^17^, ^18^, ^19^

The use of immunohistochemistry (IHC) for the determination of cancer biomarkers is a mature and powerful technology. IHC is readily available in pathology laboratories, which is relatively easy to perform and assess and can provide clinically meaningful results very quickly.^20^ In addition to IHC, there are a variety of other techniques for the diagnosis of tumors.^21^, ^22^, ^23^

As an important oncogene, the level of CCND1 in ccRCC and the role of CCND1 in the recurrence of ccRCC have not been reported. In this study, we examined the mRNA and protein levels of CCND1 in ccRCC tissues using Gene Expression Omnibus (GEO) databases, The Cancer Genome Atlas (TCGA) databases and IHC, and explored its potential value as a prognostic and recrudescent biomarker for ccRCC.

## 2 MATERIALS AND METHODS

### 2.1 Extraction of clinical and microarray gene expression data from ccRCC patient datasets

The clinical and microarray datasets of ccRCC patients were extracted from GEO database (http://www.ncbi.nlm.nih.gov/geo/) and TCGA database (http://www.cbioportal.org/data_sets.jsp). Seven microarray gene expression datasets of ccRCC patient with more than 700 specimens, GSE46699, GSE40435, GSE66272, GSE15641, GSE53757, GSE14994, and GSE36895, were obtained from GEO database.^24^, ^25^, ^26^, ^27^, ^28^, ^29^, ^30^ One microarray gene expression and clinical datasets of ccRCC patient with more than 600 specimens were obtained from TCGA database. Firstly, the probe ID was converted into a gene symbol. When different probe IDs were mapped to the same gene, the average genic expression value was calculated as the genic expression value. And then, the genic expression value was translated into log2 logarithms, and the median normalization was performed by the robust multichip averaging method.^31^ We compared the clinical specimens of ccRCC vs adjacent normal datasets using Student's t test to generate a *P* value. A *P* value < 0.05 was considered statistically significant.

The prognostic values of the CCND1 mRNA level for ccRCC patients were obtained from the TCGA database. The survival analyses were performed using the cutoff values of median CCND1 level in ccRCC patients. According to the median value of gene expression, samples were divided into high expression group and low expression group.

### 2.2 Patients and specimens

The research was composed of 202 samples from 101 ccRCC patients, who had a renal resection at the Fujian Provincial Hospital between January 2016 and June 2017. The standard requirements for patients included in the study were as follows: (a) histologically proven ccRCC; (b) no history of other malignancy tumor; (c) no prior neoadjuvant chemotherapy. The study was performed with the approval of the Ethics Committee of Fujian Provincial Hospital and complied with the Helsinki Declaration. Written informed consent was obtained from all patients involved, and specimens were stored in the hospital database and used for research.

### 2.3 IHC staining

IHC staining analysis was performed to measure the CCND1 protein level in 101 ccRCC tissues and 101 adjacent normal renal tissues. Sections of paraffin‐embedded ccRCC tissue and adjacent normal renal tissue (3 μm) were deparaffinized with dimethylbenzene and rehydrated. The sections were submerged in 0.01 mol/L sodium citrate buffer (autoclaved at 121°C for 2 minutes, pH 6.0). They were then blocked by incubation in 3% hydrogen peroxide for 10 minutes at room temperature, followed by washes with PBS solution and subsequent blocking with 10% goat serum (ZhongShan Biotechnology, China) for 30 minutes. After washing, tissue sections were incubated with anti‐CCND1 (ab61758, 1:100 dilution, Abcam, polyclonal) overnight at 4°C. The sections were then washed in PBS solution three times and incubated with HRP‐conjugated secondary antibody for 30 minutes at room temperature. Finally, tissue sections were counterstained with diaminobenzidine solution and 20% hematoxylin and dehydrated.^32^

### 2.4 Evaluation of Immunostaining Intensity

The sections stained immunohistochemically for CCND1 protein were reviewed under microscope and separately scored by two independent pathologists blinded to the clinical parameters. For CCND1 assessment, staining intensity was scored as 0 no staining, 1 weak staining, 2 moderate staining, or 3 strong staining and staining extent was scored as 1 (<25%), 2 (26%‐50%), 3 (51%‐75%), and 4 (>75%). The final score was calculated by multiplication of these two variables. Staining was graded in a five‐grade classification as follows: 0 (0 score), 1 (1‐2 scores), 2 (3‐4 scores), 3 (6‐8 scores), and 4 (>8 scores).

### 2.5 Statistical analysis

The comparison between cancer tissues and normal renal tissues was conducted using Student's t test. Kaplan‐Meier method was applied to calculate the survival analysis using IBM SPSS version 19.0. Multivariate survival analysis was performed using stepwise Cox proportional hazards regression model. Receiver operating characteristics (ROC) curve analysis was performed to analyze the ability of CCND1 as a biomarker for ccRCC recurrence prediction. R software (version 3.2.0) was utilized to develop the nomograms. All *P* values < 0.05 were considered statistically significant.

## 3 RESULTS

### 3.1 The levels of CCND1 were up‐regulated in ccRCC

**Figure 1:**
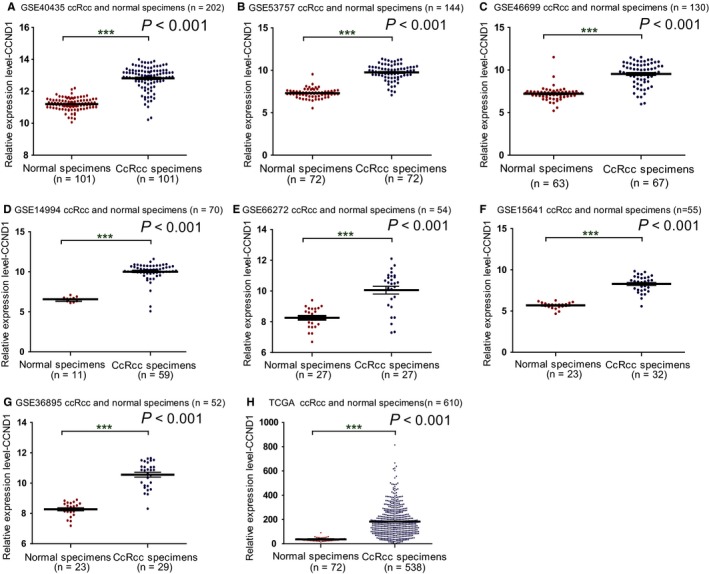
The mRNA level of CCND1 in ccRCC based on Gene Expression Omnibus and TCGA database. The mRNA levels of CCND1 in ccRCC tissues and adjacent normal renal tissues were compared. Eight mRNA datasets were employed including GSE40435 (A), GSE53757 (B), GSE46699 (C), GSE14994 (D), GSE66272 (E), GSE15641 (F), GSE36895 (G), and TCGA database (H). Abbreviations: CCND1, Cyclin‐D1; ccRCC, clear cell renal cell carcinoma; TCGA, The Cancer Genome Atlas. ^***^ *P* < 0.001

In order to explore the expression of CCND1 in ccRCC patients, a total of 7 related GEO datasets (GSE46699, GSE40435, GSE66272, GSE15641, GSE36895, GSE14994, GSE53757) (Table S1) were employed. The mRNA levels of CCND1 were higher in ccRCC tissues compared with adjacent normal renal tissues (*P* < 0.001; Figure 1A‐G). In the meanwhile, the mRNA levels of CCND1 were also up‐regulated in ccRCC tissues compared with normal renal tissues in TCGA database (*P* < 0.001; Figure 1H) (Table S2).

**Figure 2:**
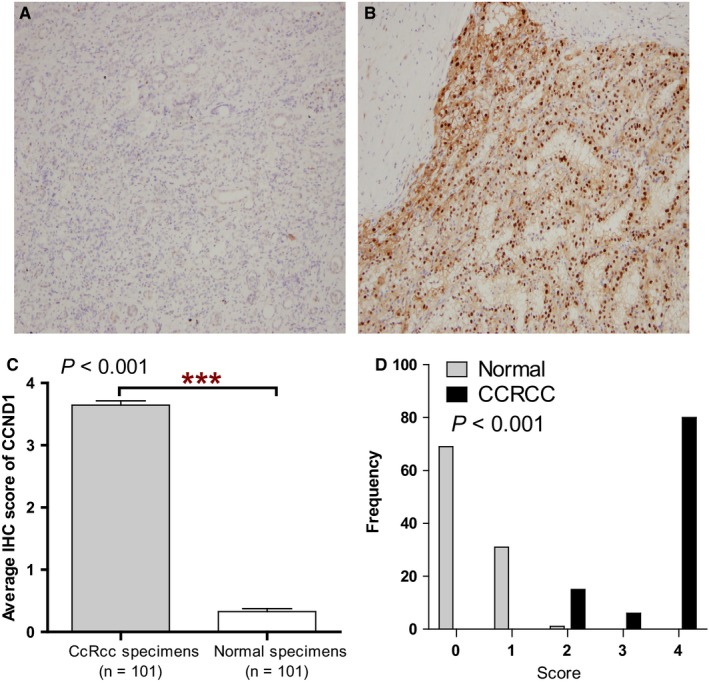
The protein level of CCND1 in ccRCC. The protein levels of CCND1 in ccRCC tissues and their adjacent normal renal tissues were compared using IHC staining. Representative adjacent normal renal tissues staining (A), ccRCC tissues staining (B), frequency distributions of proteins expression across the cohort (C), and the average scores of IHC staining (D) were shown. Abbreviations: CCND1, Cyclin‐D1; ccRCC, clear cell renal cell carcinoma; IHC, immunohistochemical. ^***^ *P* < 0.001

Next, IHC staining was used to analyze the protein levels of CCND1 in 101 ccRCC tissues and their adjacent normal renal tissues. Representative staining and the frequency distributions of the IHC scores were presented (*P* < 0.001; Figure 2A‐C). The mean scores of CCND1 proteins in ccRCC and adjacent normal renal tissues were 3.64 and 0.33, respectively (Figure 2D).

### 3.2 Decreased mRNA level of CCND1 was an unfavorable prognostic factor in ccRCC

**Figure 3:**
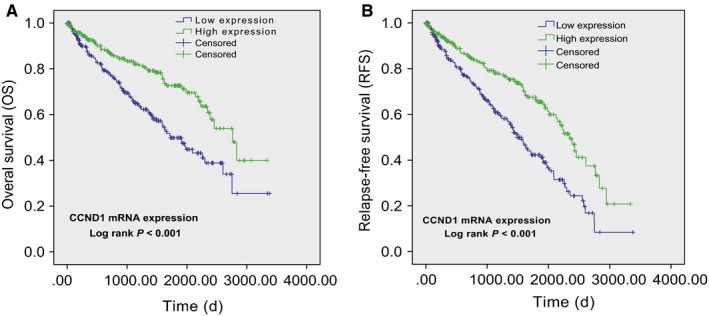
The prognostic value of CCND1 in ccRCC. The Kaplan‐Meier survival analyses of the overall survival (A) and recurrence‐free survival (B) of ccRCC patients based on their CCND1 mRNA levels in tumor tissues from TCGA database. Abbreviations: CCND1, Cyclin‐D1; ccRCC, clear cell renal cell carcinoma; TCGA, The Cancer Genome Atlas

Using TCGA database, we discovered that ccRCC patients with low mRNA level of CCND1 or high grade (grade 3 and 4) were at significantly higher risk of death. ccRCC patients with laterality = left or age > 55 also had a higher risk of death (Table 1). After adjusting for age, sex, race, and tumor grade, to our surprise, low mRNA level of CCND1 still correlated with a higher risk of death in ccRCC patients (Table 2). Kaplan‐Meier analysis showed that the ccRCC patients with low mRNA level of CCND1 had an unfavorable outcome in terms of overall survival (Figure 3A) and recurrence‐free survival (Figure 3B).

**Table 1:** Univariate analysis of the correlation between clinicopathological parameters and survival of ccRCC patients in TCGA cohort

Variables	Patients (n)	MST (d)	Log‐rank test	*P*
Age (y)				
≤55	182	NA		
>55	345	1986	11.178	0.001*
Gender				
Female	185	2343		
Male	342	2299	0.098	0.755
Laterality				
Left	248	2227		
Right	278	NA	5.517	0.019*
Race				
White	456	2343		
Other	63	1913	0.424	0.515
Tumor stage				
I/II	316	2764		
III/IV	200	1200	87.73	0.000*
Tumor grade				
1/2	231	2752		
3/4	277	1724	33.388	0.000*
Tumor recurrence				
Recurrence	138	1034		
Without recurrence	351	NA	116.865	0.000*
CCND1 expression				
Low	263	2764		
High	263	1912	14.992	0.000*

Abbreviations: CCND1, Cyclin‐D1; ccRCC, clear cell renal cell carcinoma; MST, Median survival time; TCGA, The Cancer Genome Atlas.

* *P* < 0.05.

**Table 2:** Multivariate analysis of the correlation between clinicopathological parameters and survival of ccRCC patients in TCGA cohort

Covariates	SE	HR	95% CI for HR	*P*
Age (≤55 vs > 55)	0.191	0.605	0.416‐0.879	0.080
Laterality (left vs right)	0.163	1.487	1.080‐2.046	0.015*
Tumor grade (1/2 vs 3/4)	0.195	1.440	0.728‐2.847	0.000*
CCND1 (low vs high)	0.175	1.757	1.247‐2.477	0.001*

Abbreviations: CCND1, Cyclin‐D1; TCGA, The Cancer Genome Atlas.

* *P* < 0.05.

### 3.3 The mRNA level of CCND1 was not associated with the prognosis of cRCC and pRCC

**Figure 4:**
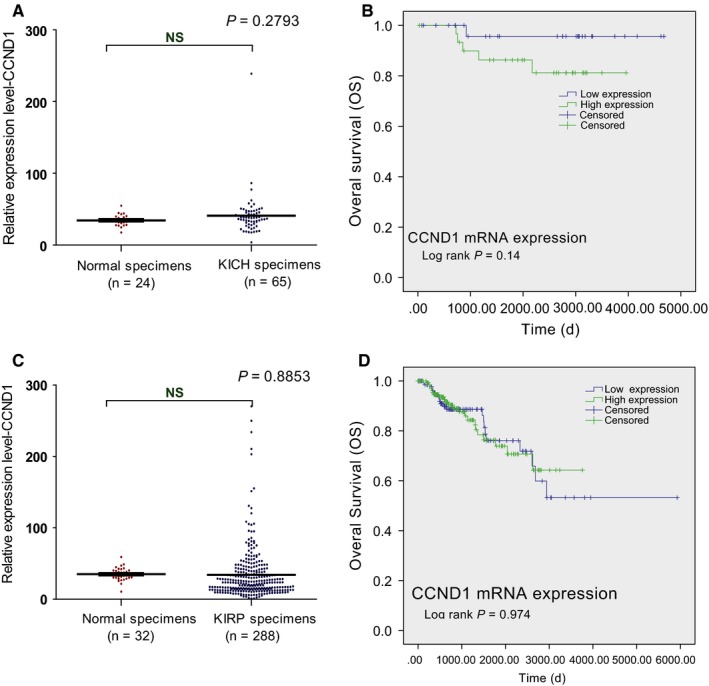
The mRNA level and prognostic value of CCND1 in cRCC and pRCC based on TCGA database. The mRNA levels of CCND1 in cRCC tissues and adjacent normal renal tissues were compared (A). The Kaplan‐Meier survival analyses of the overall survival of cRCC patients based on their CCND1 mRNA levels in tumor tissues from TCGA database (B). The mRNA level of CCND1 in pRCC tissues and adjacent normal renal tissues was compared (C). The Kaplan‐Meier survival analyses of the overall survival of pRCC patients based on their CCND1 mRNA levels in tumor tissues from TCGA database (D). Abbreviations: CCND1, Cyclin‐D1; cRCC, chromophobe renal cell carcinoma; pRCC, papillary renal cell carcinoma; TCGA, The Cancer Genome Atlas

In order to investigate whether CCND1 is associated with worse prognosis of all types of RCC, the CCND1 mRNA levels in cRCC and pRCC tissues were compared with their respective adjacent renal tissues using TCGA database and no difference was observed (*P* > 0.05; Figure 4A,C). With the cutoff value set at the median, we found that the mRNA level of CCND1 was not associated with the clinical outcomes of cRCC or pRCC patients (*P* > 0.05; Figure 4B,D), suggesting CCND1 level is specifically associated with prognosis of ccRCC.

### 3.4 The mRNA level of CCND1 correlated with tumor grade in ccRCC

**Figure 5:**
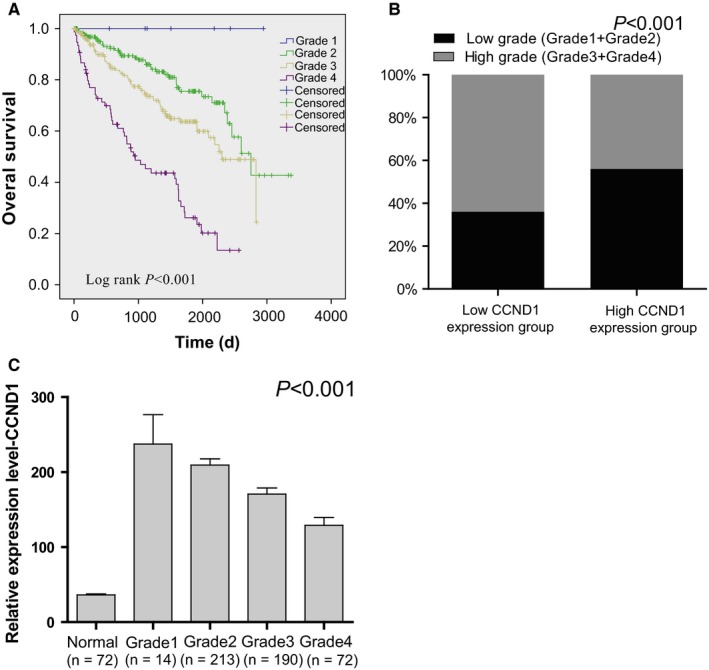
The correlation between CCND1 and tumor grade in ccRCC. The Kaplan‐Meier survival analyses of tumor grades in ccRCC (A). A histogram showing the percentage of tumor grades in high/low CCND1 level of ccRCC specimens (B). The mRNA level of CCND1 in different grades of ccRCC specimens was compared (C). Abbreviations: CCND1, Cyclin‐D1; ccRCC, clear cell renal cell carcinoma

The survival time of ccRCC patients is closely associated with tumor grade (*P* < 0.001; Figure 5A), and previous studies have reported that the level of CCND1 is related to tumor grade in some cancers.^15^, ^33^, ^34^, ^35^ Therefore, using the median expression as the cutoff point, we tested the proportion of different tumor grades in the CCND1 low and high expression groups. In total, 56% patients with CCND1 high expression contained low‐grade tumor group, but only 36% patients with CCND1 low expression contained low‐grade tumor (chi‐square test, *P* < 0.001; Figure 5B), indicating CCND1 mRNA level decreases with increasing ccRCC tumor grades (*P* < 0.001; Figure 5C).

### 3.5 Decreased mRNA level of CCND1 was related to tumor recurrence in ccRCC

**Figure 6:**
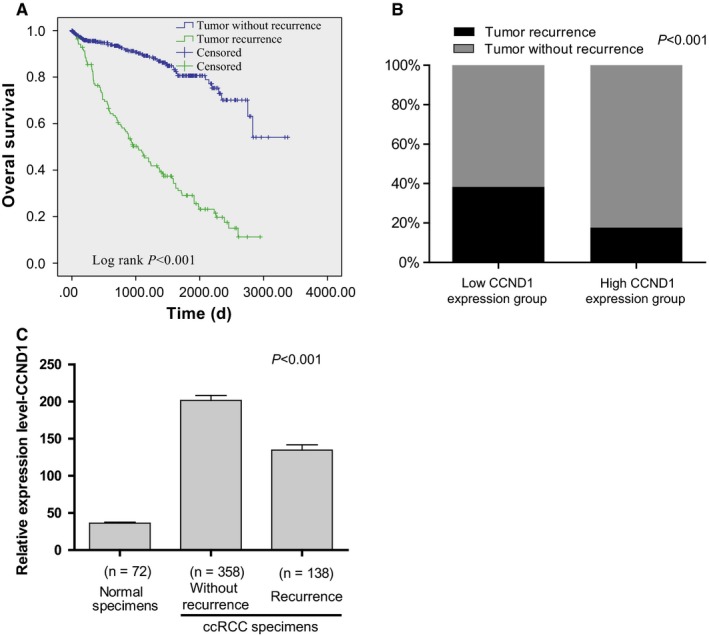
The relation between CCND1 and tumor recurrence in ccRCC. The Kaplan‐Meier survival analyses of tumor recurrence in ccRCC (A). A histogram showing the percentage of tumor recurrence in high/low CCND1 level of ccRCC specimens (B). The mRNA level of CCND1 in tumor recurrence and tumor without recurrence in ccRCC was compared (C). Abbreviations: CCND1, Cyclin‐D1; ccRCC, clear cell renal cell carcinoma

Tumor recurrence is an important adverse factor related to short survival time. We observed that patients with tumor recurrence were at significantly increased risk of death in ccRCC (Figure 6A). Meanwhile, using the median expression as the cutoff point, we tested the probability of tumor recurrence in the CCND1 low and high expression groups. In total, 38% patients with CCND1 low expression had recurrence, but only 17% patients with CCND1 high expression had recurrence (chi‐square test, *P* < 0.001; Figure 6B). At the same time, we found that the level of CCND1 is higher in the patients without tumor recurrence compared to those patients with tumor recurrence (*P* < 0.001; Figure 6C). These results suggested that the level of CCND1 was associated with tumor recurrence in ccRCC.

### 3.6 The mRNA level of CCND1, combined with tumor grade, was able to better predict tumor recurrence in ccRCC patients

**Figure 7:**
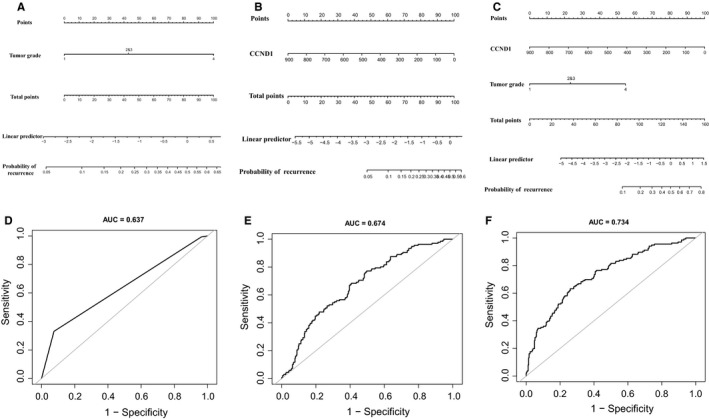
The role of CCND1 and tumor grade in predicting tumor recurrence in ccRCC. Nomograms for the role of tumor grade in predicting ccRCC tumor recurrence (A). Nomograms for the role of CCND1 in predicting ccRCC tumor recurrence (B). Nomograms for the role of tumor grade and CCND1 in predicting ccRCC tumor recurrence (C). ROC analysis of the tumor grade for ccRCC recurrence risk prediction (D). ROC analysis of CCND1 for ccRCC recurrence risk prediction (E). ROC analysis of CCND1 and tumor grade for ccRCC recurrence risk prediction (F). Abbreviations: CCND1, Cyclin‐D1; ccRCC, clear cell renal cell carcinoma; ROC, receiver operating characteristics

Our results indicated that the level of CCND1 and tumor grade may be related to tumor recurrence in ccRCC patients. Therefore, nomograms for the prediction of recurrence probabilities, which included CCND1 and tumor grade, were constructed (Figure 7). ROC curve was used to analyze the ability of CCND1 and tumor grade to discriminate between ccRCC patients with or without recurrence. According to the ROC analysis, the area under the curve (AUC) of the nomograms for recrudescent probability based on tumor grade (Figure 7D) and CCND1 (Figure 7E) was 0.637 and 0.674, respectively. The AUC of combined CCND1 and tumor grade was 0.734 (Figure 7F), suggesting CCND1 can help distinguish potential recrudescent patients.

## 4 DISCUSSION

The development of ccRCC is complex and affected by factors other than pathologic factors. Integrated prognostic algorithms are consequently needed to better predict patient outcomes. Currently, some risk assessment models were used to predict the postoperative risk for ccRCC patients. However, these models mainly focus on the pathological characteristics and ignore the components of genetic characters which also play an important role in tumor progression. It is reasonable to combine gene expression and pathologic factor to establish predictive models.

Univariate analysis revealed that CCND1 was not only associated with prognosis, but also tumor grade and tumor recurrence. Previous studies have shown that high expression of CCND1 was associated with poor differentiation in oral squamous cell carcinoma, gastric cancer, laryngeal squamous cell carcinoma, and medulloblastoma or displayed no significant associations with cell differentiation in endometrial carcinoma.^36^, ^37^, ^38^, ^39^, ^40^ However, the level of CCND1 decreased with the increase in ccRCC grades. These results indicated that the poorer the cell differentiation, the lower the level of CCND1 in ccRCC.

In this study, high mRNA levels of CCND1 were observed in ccRCC tissues compared with normal renal tissues using GEO database and TCGA database. At the same time, increased protein levels of CCND1 were observed in 101 ccRCC tissues compared with their adjacent normal renal tissues using IHC. These results suggested that the expression of CCND1 was up‐regulated in ccRCC. Usually, a gene aberrantly highly expressed in cancer tissues acts as a negative prognostic factor.^41^, ^42^ As an oncogene, increased expression of CCND1 has been proven to be an unfavorable prognostic factor in some cancers including gastric adenocarcinoma patients, lung adenocarcinoma patients, oropharyngeal cancer, and pancreatic carcinoma.^15^, ^33^, ^34^, ^35^ However, in breast cancer, the increased level of CCND1 has been linked to favorable prognosis.^43^ These results suggested that CCDNI plays a variety of roles in different cancers. In this study, the increased mRNA level of CCND1 is a favorable prognostic factor in ccRCC. Previous study has shown that the levels of CCND1 mRNA were up‐regulated in the RCCs of Saudi Arabian patients,^44^ which is consistent with our study on Chinese population. However, in the other two types of RCC, cRCC and ccpRCC, we found no difference in CCND1 expression and prognosis, suggesting CCND1 may play a different role in ccRCC compared with other types of RCC cancer.

Previous studies have shown that patients bearing tumors with high amplification of CCND1 had an increased risk of recurrence for invasive breast cancer and high expression of CCND1 predicts recurrence in supratentorial ependymomas.^19^, ^45^ Using the median expression as the cutoff point, we tested the probability of tumor recurrence in ccRCC patients with low and high expression of CCND1. However, we found that patients with high CCND1 expression had a lower risk of tumor recurrence in ccRCC compared to those with low CCND1 expression. The poor prognosis of patients with low level of CCND1 may be due to the higher recurrence rate. Therefore, a nomogram was established based on tumor grade and CCND1. Notably, our nomograms showed that CCND1 is a strong determinant for prediction of recurrence. The patients with high CCND1 level appear to have a more favorable prognosis because they present more frequently with low‐grade tumors and low rate of recurrence. The nomogram may be useful for patient counseling as it helps predict the rate that ccRCC patients will encounter recurrence.

## 5 CONCLUSION

In conclusion, to the best of our knowledge, this is the first report on CCND1 expression in ccRCC. This study demonstrated that higher levels of CCND1 mRNA and protein were observed in ccRCC compared to their adjacent tissues. Different from many other types of cancer, the decreased expression of CCND1 in ccRCC acts as a favorable prognostic factor, which could be integrated with tumor grade to generate a nomogram to predict recurrence risk for ccRCC patients. The patients with low CCND1 level should be guided to shortened follow‐up interval and increased follow‐up times. The molecular mechanisms underlying why the high mRNA level of CCND1 contributing to favorable prognosis of ccRCC are still unclear. Further investigation is needed to elucidate this observation.

## CONFLICT OF INTEREST

The authors declare no conflict of interest.
